# Description of *Babesia galileei* sp. nov. A piroplasmid species causing severe disease in domestic cats

**DOI:** 10.1186/s13071-024-06371-w

**Published:** 2024-07-09

**Authors:** Gad Baneth, Yaarit Nachum-Biala, Ann Dvorkin, Irit Arogeti, Shlomo Amiel, Yamit Soueid, Dor Shwartz, Kosta Y. Mumcuoglu, Harold Salant

**Affiliations:** 1 Koret School of Veterinary Medicine, The Hebrew University, Rehovot, Israel; 2 Rehovot Veterinary Hospital, Rehovot, Israel; 3 Vetmed LTD, Shavei Tzion, Israel; 4 Department of Microbiology and Molecular Genetics, The Hebrew University-Hadassah Medical School, Jerusalem, Israel

**Keywords:** *Babesiafelis*, Babesiosis, Domestic cat, *Haemaphysalis adleri*, Israel

## Abstract

**Background:**

Babesiosis is a tick-borne infection caused by piroplasmid protozoa and associated with anemia and severe disease in humans, domestic animals and wildlife. Domestic cats are infected by at least six *Babesia* spp. that cause clinical disease.

**Methods:**

Infection with a piroplasmid species was detected by microscopy of stained blood smears in three sick cats from Israel. Genetic characterization of the piroplasmid was performed by PCR amplification of the *18S rRNA*, cytochorme B (*CytB*) and heat shock protein 70 (*HSP70*) genes and the internal transcribed spacer (ITS) locus, DNA sequencing and phylogenetic analysis. In addition, *Haemaphysalis adleri* ticks collected from two cats were analyzed by PCR for piroplasmids.

**Results:**

The infected cats presented with anemia and thrombocytopenia (3/3), fever (2/3) and icterus (1/3). Comparison of gene and loci sequences found 99–100% identity between sequences amplified from different cats and ticks. Constructed phylogenetic trees and DNA sequence comparisons demonstrated a previously undescribed *Babesia* sp. belonging to the *Babesia* sensu stricto (clade X). The piroplasm forms detected included pear-shaped merozoite and round-to-oval trophozoite stages with average sizes larger than those of *Babesia felis*, *B. leo* and *B. lengau* and smaller than canine *Babesia* s.s. spp. Four of 11 *H. adleri* adult ticks analyzed from cat # 3 were PCR positive for *Babesia* sp. with a DNA sequence identical to that found in the cats. Of these, two ticks were PCR positive in their salivary glands, suggesting that the parasite reached these glands and could possibly be transmitted by *H. adleri*.

**Conclusions:**

This study describes genetic and morphological findings of a new *Babesia* sp. which we propose to name *Babesia galileei* sp. nov. after the Galilee region in northern Israel where two of the infected cats originated from. The salivary gland PCR suggests that this *Babesia* sp. may be transmitted by *H. adleri*. However, incriminating this tick sp. as the vector of *B. galilee* sp. nov. would require further studies.

**Graphical Abstract:**

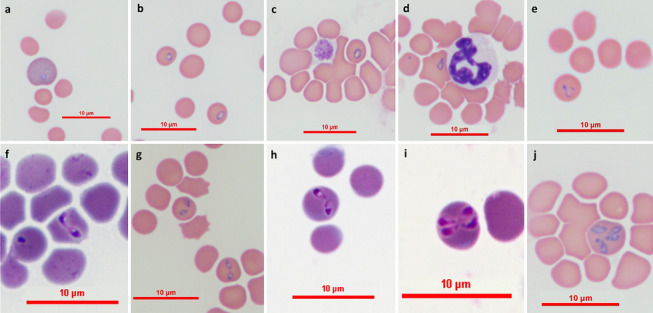

**Supplementary Information:**

The online version contains supplementary material available at 10.1186/s13071-024-06371-w.

## Background

*Babesia* Starcovici, 1893, is a tick-borne protozoan genus from the phylum Apicomplexa, class Piroplasmea, order Piroplasmida. *Babesia* spp. infect domestic and wildlife animals and humans and cause severe hemolytic disease which could eventually lead to a fatal outcome [1]. More than 100 different species of *Babesia* have been described in domestic mammals and wildlife as well as in some avian species, and *Babesia* spp. are the second most frequently reported blood parasites of mammals after trypanosomes [2]. New *Babesia* spp. have been described morphologically and molecularly characterized in a variety of wildlife and domestic vertebrates in the past 20 years [1]. Domestic cats are infected with clinical disease by several *Babesia* spp. and sub-species which have been characterized genetically and described morphologically (Table 1). These include: (i) *Babesia felis* Davis, 1929, initially described from a wild cat (*Felis ocreata*) and then from domestic cats [3–5]*;* (ii) *Babesia canis presentii* Baneth et al., 2004 [6]; (iii) *Babesia leo* Penzhorn et al., 2001, initially described in lions (*Panthera leo*) and then from cats [7–9]; (iv) *Babesia hongkongensis* [10]; (v) *Babesia lengau* Bosman et al., 2010, initially described in cheetahs (*Acinonyx jubatus*) and thereafter from cats [11, 12]; and (vi) *Babesia panickeri* Panicker et al., 2020 [13]. In addition, *Babesia* sp. Cat Western Cape was reported to cause disease in cats in South Africa but it has not been described as a new species yet [8, 14].

**Table 1:** *Babesia* spp. described morphologically from domestic cats and wildlife felines with comparison of life stage sizes

*Babesia* species	Size in micrometers	Reference
*Babesia galileei* sp. nov	Merozoites 1.70 ± 0.37 (range 0.81–3.05) × 0.91 ± 0.25 (range 0.54–1.39) Trophozoites 1.53 ± 0.35 (range 0.81–2.50) × 1.07 ± 0.25 (range 0.54–1.62)	Current
*Babesia felis*	1.25 (range 1–2.25)	[3]
*Babesia leo*	Only trophozoites were measured 1.05 ± 0.22 (range 0.63–1.73) × 1.0 ± 0.14 (range 0.62–1.03)	[9]
*Babesia lengau*	Small ovoid forms 1.3 ± 0.2 × 1.3 ± 0.2 (range 0.8–1.8); Dividing trophozoites (to merozoites) 1.91 ± 0.2 × 1.1 (range 1.6–2.3 and 0.6–1.3)	[11]
*Babesia hongkongensis*	Only ring forms were measured Ring form (trophozoites) 1.4–1.6	[10]
*Babesia canis* subsp. *presentii*	Merozoites 2.5 ± 0.36 (range 1.3–2.9) × 1.4 × 0.3 (range 0.7–2.3); Trophozoites 2.7 ± 0.42 (range 1.8–3.2) × 1.7 ± 0.38 (range 1.2–2.5)	[6]
*Babesia panickeri*	Only merozoites are mentioned Merozoites 2.7 ± 0.3 (1.9–3.6) × 1.3 ± 0.21 (1.02–1.7)	[13]
*Babesia* sp. lynx genotype	Merozoites 1.9 ± 0.37 (range 1.19–2.79) × 1 ± 0.18 (range 0.72–1.75) Trophozoites 1.87 ± 0.41 (range 1.33–284) × 1.55 ± 0.31 (range 0.95–2.14)	[30]

Additional *Babesia* spp. described to infect cats include *Babesia cati* reported from an Indian wild cat (*Felis catus*) [15], *Babesia pisicii* reported from European wild cats (*Felis silvestris*) [16] and some *Babesia* spp. which are typically found in canines and have mostly been detected only by PCR and reported based on molecular data including *Babesia canis* [17], *B. vogeli* [18], *B. gibsoni* [19] and *B. vulpes* [17]. DNA of *Babesia microti*, which infects rodents and humans, has also been reported in cat surveys from Europe and Pakistan [20, 21].

This study describes severe clinical babesiosis which was detected in cats brought for veterinary care in Israel. Initial PCR and genetic analysis of the infecting organism suggested that the cats are infected with a previously unknown *Babesia* sp. The aim of this study was to morphologically describe, genetically characterize and record the clinical disease associated with this piroplasmid organism in domestic cats.

## Methods

### Animal samples

Piroplasm parasites were detected in erythrocytes by light microscopy in blood smears stained by Romanowsky staining solutions in three domestic cats from Israel whose blood was tested at the laboratory for vector-borne diseases, Koret School of Veterinary Medicine, in Rehovot, Israel, during the years 2008–2023. The medical history, physical examination findings and complete blood count (CBC) as well as other diagnostic and laboratory test results were extracted from the medical records of all cats (Table 2). Blood smears were visualized by oil immersion microscopy at 1000 × magnification, and parasite sizes were measured using a micrometer.

**Table 2:** Demographic and clinical characteristics of cats infected with *Babesia galileei* sp. nov. included in the study

Cat number	1	2	3
Location in Israel	Kibbutz Yehiam	Holon	Harashim
Year of diagnosis	2008	2022	2023
Sex, age (years)	Male castrated; 3	Male castrated; 11	Male castrated; 11
Breed	DSH	DSH	DSH
Fever	+ ; 40.5 °C	+ ; 40.7 °C	−; 37.2 °C
Lethargy	+	+	+
Anorexia	+	+	+
Pale mucous membranes	+	+	+
Icterus	–	–	+
Anemia; hematocrit; MCV (fl); MCHC (g/l)	+ ; 13.3; 36.4; 38.9	+ ; 23; 49; 38.2	+ ; 18.5; 39.6; 34.6
Leukocytosis; WBC (× 10^6^/ul)	−; 12.36	−; 7.85	+ ; 35.9
Thrombocytopenia; platelet count (× 10^6^/ul)	+ ; 90,000	+ ; 79,000	+ ; 68,000
Co-infection			
FIV/FeLV	+/-	-/-	-/-
Hemotrophic *Mycoplasma*	–	–	*Candidatus* Mycoplasma haemominutum +
Treatment	Imidocarb dipropionate 3.5 mg/kg subcutaneously	Imidocarb dipropionate 3.5 mg/kg subcutaneously	Imidocarb dipropionate at 2.5 mg/kg subcutaneously

*DSH* domestic short-haired, rectal body temperature reference interval (RI) 38.1–39.2 ºC; mean cell volume (MCV) RI 41.3–52.6 fL; mean corpuscular hemoglobin concentration (MCHC) RI 27–32.8, white blood cells (WBC) RI 6.3–19.6 × 10^3^/ul, platelet numbers RI 156–626 × 10^3^/ul

### Molecular detection of *Babesia* and other pathogens in animal samples

DNA was extracted from 200 μl blood of EDTA anticoagulated blood using the Qiagen DNeasy Blood & Tissue Kit (Qiagen, USA) following the manufacturer’s protocol. *Babesia* spp. molecular characterization was done using several PCRs with primers, and conditions are specified in Table 3. PCR was performed in a total volume of 25 μl using the PCR-ready High Specificity mix (Syntezza Bioscience, Jerusalem) with 4 μl of DNA template, 400 nM of each primer and sterile DNase/RNase-free water (Sigma, St. Loius, MO, USA). Amplification was performed by a programmable conventional thermocycler (Biometra, Goettingen, Germany). Initial denaturation at 95 °C for 5 min was followed by 35–45 cycles of denaturation at 95 °C for 30 s, annealing and extension conditions (Table 3) and final extension at 72 °C for 30 s, unless detailed otherwise. The extension step was continued, after the last cycle, the for an additional 5 min. PCR products were electrophoresed on 1.5% agarose gels stained using ethidium bromide and evaluated for the size of amplified fragments under UV light by comparison to a 100-bp DNA molecular weight marker. Positive DNA controls from naturally infected dogs with *B. vogeli* were run with each corresponding reaction. Non-template control (NTC) reactions were performed by using the same reagents and procedures described above but without DNA added to the reaction to rule out contaminations. PCR was also used for testing the cat samples for infection with *Mycoplasma* [17], *Hepatozoon*, *Ehrlichia* and *Anaplasma* spp. (Table 4). Samples were also tested for the presence of antibodies for the feline immunodeficiency virus (FIV) and for feline leukemia virus (FeLV) antigenemia using a commercial assay (SNAP FIV/FeLV Combo Test, IDEXX Laboratories, Westbrook, ME, USA).

**Table 3:** List of primers used for characterization of *Babesia* spp. in this study

Target locus\gene	Primers	Primer sequence	Fragment length in base pairs	Annealing temperature and time	Number of cycles	Reference
piroplasmid* 18S rRNA*	piroplasmidd-F	CCAGCAGCCGCGGTAATTC	400	64 °C for 45 s	35	[51]
	piroplasmid-R	CTTTCGCAGTAGTTYGTCTTTAACAAATCT				
piroplasmid *18S rRNA*	Nbab 1F	AAGCCATGCATGTCTAAGTATAAGCTTTT	1700	60 °C for 40 s (followed by 72 °C for 60 s)	35	[50]
	TB Rev	AATAATTCACCGATCACTCG				
piroplasmid ITS*	ITS_F	GTGAACCTTATCACTTAAAGG	940	51 °C for 90 s	40	[33]
	ITS_R	TTCRCTCGCCGYTACT				
piroplasmid *Cytochrome B* (*CytB*)	COB-F	CCATAGCAATTAATCCAGCTA	550	54 °C for 30 s	35	[52]
	COB-R	ACCTTGGTCATGGTATTCTGG				
piroplasmid Heatshock protein 70 (Hsp70)	HSP70for	GCTATTGGTATTGACTTGGG	333	54 °C for 120 s	40	[49]
	HSP70rev	CCTTCATCTTGATAAGGACC				

^*^Amplification includes parts of the *18S rRNA*, internal transcribed spacer (ITS) 1, *5.8S rRNA*, ITS2 and partial *28S rRNA* gene

**Table 4:** List of primers used for detection of *Hepatozoon*, *Ehrlichia*, *Anaplasma* and *Mycoplasma* spp. in this study

Target locus\gene	Primers	Primer sequence	Fragment length in base pairs	Annealing temperature and time	Number of cycles	Reference
*Hepatozoon 18S rRNA*	Hepatozoon18S-F	GGTAATTCTAGAGCTAATACATGAGC	574	50 °C for 30 s	35	[53]
	Hepatozoon18S-R	ACAATAAAGTAAAAAACAYTTCAAAG				
*Ehrlichia*/*Anaplasma* *16SrRNA*	Ec 16S–F Ec 16S-R	TCGCTATTAGATGAGCCTACGT GAGTCTGGACCGTATCTCAGT	123	60 °C for 30 s	45	[54]
*Mycoplasma 16S rRNA*	HBT-F	ATACGGCCCATATTCCTACG	595	60 °C for 30 s	40	[17]
	HBT-R	TGCTCCACCACTTGTTCA				

DNA from all the positive PCR products was sequenced at the Center for Genomic Technologies, Hebrew University of Jerusalem, Israel, using the BigDye Terminator v3.1 Cycle Sequencing Kit and an ABI PRISM 3100 Genetic Analyzer (Applied Biosystems, Foster City, CA, USA). The resulting sequences were evaluated using ChromasPro software version 2. 1.1 (Technelysium Pty Ltd., Australia) and compared for similarity with sequences available in GenBank using the BLAST program (http://www.ncbi.nlm.nih.gov/BLAST/). New DNA sequences from *Babesia*-infected cats in this study were deposited as new accessions in GenBank.

The near full length of the piroplasma *18S rRNA* gene was sequenced in parts using 3.2 pmol of the following primers: Nbab1F (5′-AAGCCATGCATGTCTAAGTATAAGCTTTT-3′), TB Rev (5′-AATAATTCACCGGATCACTCG-3′), BT 2R (5′-CCCGTGTTGAGTCAAATTAAGCCG-3′), BT 3F (5′-GGGCATTCGTATTTAACTGTCAGAGG-3′), Nbab 4F (5′-CCGTTAACGGAACGAGACCTTAACC-3′) and Nbab 4R (5′-GGTAGGCCAATACCCTACCG-3′) [22]. Sequences were then reconstructed using MEGA version X (http://www.megasoftware.net) [23]. Multiple sequence alignment (MSA) was done using EMBL-EBI—CLUSTAL OMEGA [24].

### Tick samples

Ticks collected from two of the cats with babesiosis in the study (cats 1 and 3) were identified morphologically and analyzed for *Babesia* infection by PCR. Some of the ticks from cat # 3 were dissected, and their salivary glands, guts and remaining body parts were separated. DNA was extracted from ticks and their organs using a commercial kit (DNeasy Blood & Tissue Kit, Qiagen, Hilden, Germany) following the manufacturer's instructions. PCR of ticks was used to detect the presence of *Babesia* spp. DNA was analyzed using the piroplasmid PCR and primers (Table 3). All positive DNA amplicons were sequenced as detailed above and identified using the BLAST program as described for *Babesia* sp. sequences from cat blood.

### Phylogenetic analysis

Phylogenetic analyses, which included DNA sequences derived in this study, were performed to compare these sequences to other piroplasmid spp. sequences deposited previously in GenBank. Sequences were analyzed using the MEGA version X [23], and phylogenetic trees were constructed using maximum likelihood algorithms. A model for each phylogenetic tree was chosen according to the Aikaike information criterion (AIC) and was specified for each tree separately. Bootstrap replicates were done to estimate node reliability, and values were extracted from 1000 randomly selected samples of the aligned sequence data.

## Results

**Family** **Babesiidae** **Poche,** **1913**

**Genus** ***Babesia*** **Starcovici,** **1893**

***Babesia*** ***galileei*** **sp.** **nov.**

*Type-host* Domestic cat *Felis catus* Linnaeus, 1758 (Mammalia: Felidae).

*Type-locality* City of Holon (32°01′01″N 34°46′45″E), Israel.

*Other localities* Village of Harashim (32°57′24″N 35°19′41″E), Israel and Kibbutz Yehiam (32°59′49″N 35°13′15″E), Israel.

**Fig. 1:**
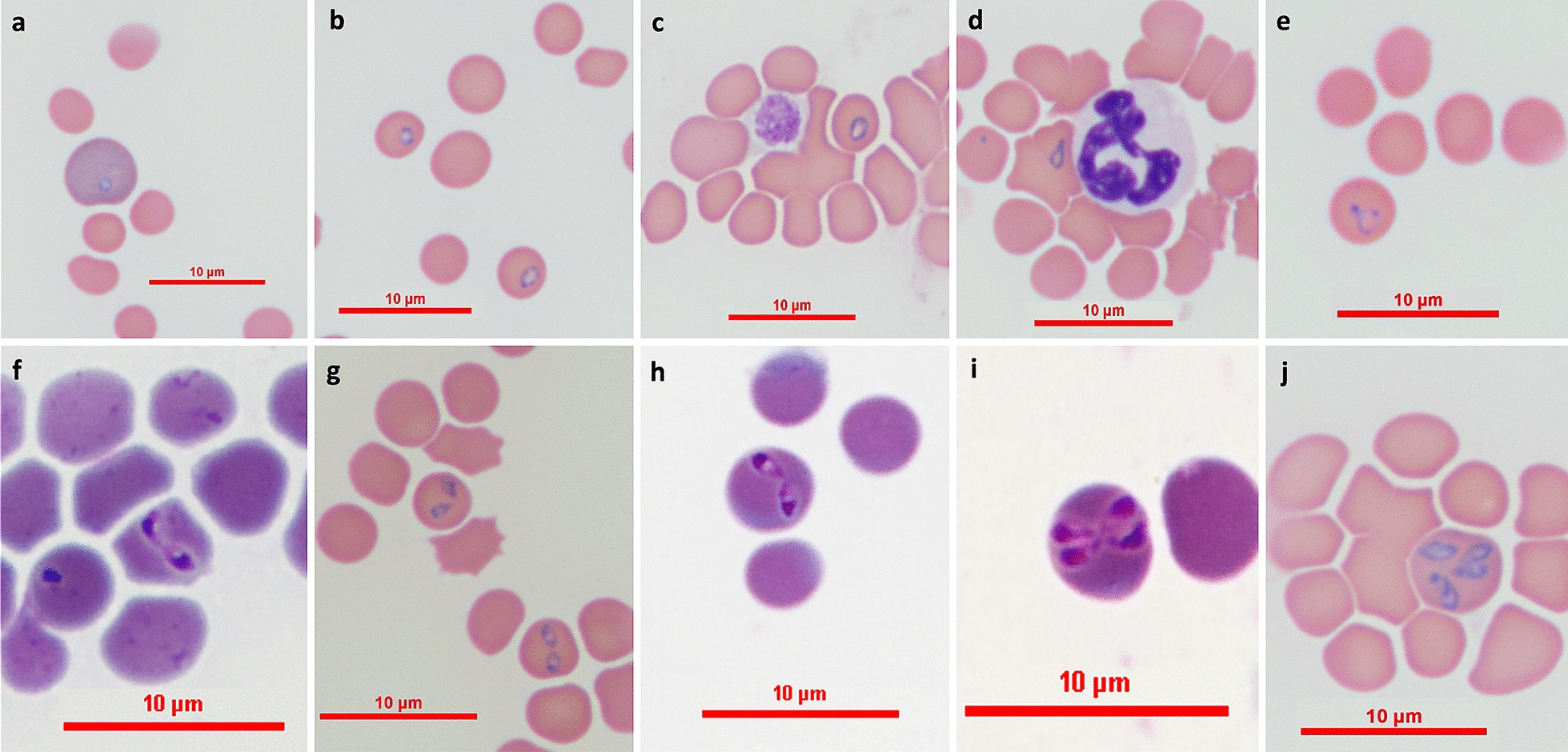
Proposed evolution of *Babesia galileei* sp. nov. development and division. Round early trophozoite form within a young erythrocyte (**a**) followed by elongating oval trophozoites (**b**, **c**). Trophozoite forms develop a pointed end (**d**) and begin to divide into merozoites creating a triangular shape (**e**), which eventually separates into two pear-shaped merozoites which are initially attached to each other (**f**). Pairs of merozoites gradually become distant from each other (**g**, **h**) and may divide again to form four merozoites within the same erythrocyte (**i**, **j**). The holotype parasite from cat # 2 is shown in Fig. 1 h. Modified Wright’s and quick Romanowsky staining

*Type-material* A stained thin blood smear from an 11-year-old Israeli male domestic cat containing the holotype (cat. no. 2; Fig. 1h) was deposited in the National Natural History Collection of the Hebrew University of Jerusalem, Israel, under accession number HUJINV 1000000006. In addition, genomic DNA extracted from the blood of infected cats no. 2 and 3 was deposited at the Koret School of Veterinary Medicine, Hebrew University of Jerusalem, Rehovot, Israel, under accession numbers 9394(P) and 2358941(C), respectively.

*Vector* Unknown. The ixodid tick *Haemaphysalis adleri* Feldman-Muhsam, 1951 [25] is suspected.

*Representative DNA sequences* Present study (GenBank: PP620719-PP62021: 18S *rRNA*; PP620716-PP620718: ITS; PP24317, PP590338, PP590339: *CytB*; PP624315, 624316; HSP 70).

*ZooBank registration* To comply with the regulations set out in article 8.5 of the amended 2012 version of the International Code of Zoological Nomenclature (ICZN) [26], details of the new species were submitted to ZooBank. The Life Science Identifier (LSID) of the article is urn:lsid:zoobank.org:pub:98EBB21B-88F0-4385-9D64-663DC0E8B975. The LSID for the new name *Babesia galileei* is urn:lsid:zoobank.org:act:3751D81F-5191-4986-A69D-5D70816E5E65.

*Etymology* The species is named after the Galilee district in northern Israel where the first cat infected with this parasite was detected.

## Description

All measurements are in micrometers ± standard error.

*Trophozoites* (Measurements based on 84 parasites; see Fig. 1a, d.) Round to oval forms measuring 0.81–2.50 (1.53 ± 0.35) in length and 0.54–1.62 (1.07 ± 0.25) in width (*n* = 84) with basophilic-staining nuclear material adhering to the parasite’s outer limits. As the trophozoites develop they become more oval and eventually develop a pointed end (Fig. 1d) after which they begin to divide into merozoites creating a triangular shape (Fig. 1e), which eventually separates into two pear-shaped merozoties which are initially attached to each other (Fig. 1f).

**Fig. 2:**
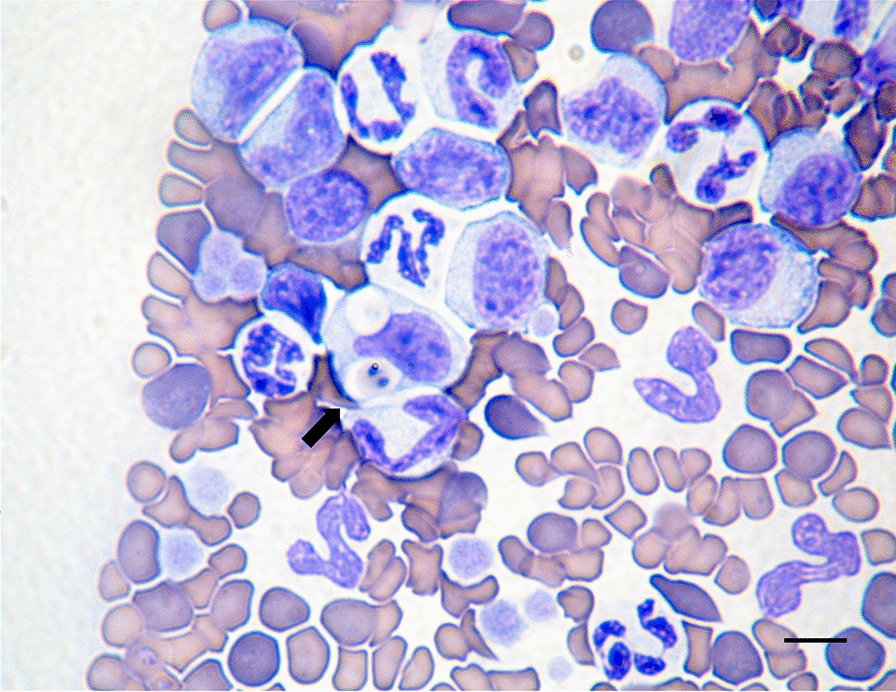
Two merozoites of *Babesia galileei* sp. nov. in a phagocytosed erythrocyte within a monocyte. The parasites are present in a monocyte (arrow) at the edge of a blood smear from cat # 1. Modified Wright’s stain. Bar = 5 microns

*Merozoites* (Measurements based on 108 parasites; see Fig. 1f, j.) Pear-shaped merozoites with eccentric nuclei presenting as two (Fig. 1f–h) or four intraerythorocytic parasites (Fig. 1i, j). The merozoites are found in different stages of development measuring 0.81–3.05 (1.70 ± 0.37) in length and 0.54–1.39 (0.91 ± 0.25) in width (*n* = 108) with basophilic staining nuclei in the outer rounded poles of the piroplasm forms (Fig. 1f–h, j). Pairs of merozoites were also seen in erythrocytes that were phagocytosed by large monocytes in the feather edge of the blood smear (Fig. 2).

### Differential diagnosis

Intraerythrocytic parasites presented in several shapes. Trophozoites of *B. galileei* sp. nov. are initially small and round (Fig. 1a) and gradually enlarge and assume an oval shape (Fig. 1b, c). Trophozoite shapes that develop a pointed end (Fig. 1d) begin to divide and widen to a fan-like shape in which two early nuclei may be seen (Fig. 1e) and then complete the division to produce pear-shaped merozoites which are initially attached to each other following binary fission (Fig. 1f) and then separate and detach (Fig. 1g). Merozoites possess basophilic staining dense chromatin nuclei located in the rounded poles of their elongated forms (Fig. 1f–i). Four merozoites were found within some erythrocytes presumably after pairs of merozoites had divided, creating four merozoites which took up about half of the erythrocyte volume (Fig. 1i, j).

The forms of *B. galileei* sp. nov. described here from cat erythrocytes include pear-shaped merozoites that present in pairs with their pointed ends touching or close to each other, similar to the forms of large *Babesia* spp. of dogs such as *B. vogeli* Reichenow, 1937, *Babesia canis* Pianna & Galli-Vallerio, 1895, and *Babesia rossi* (Nuttal, 1910) Wenyon, 1926. However, they are much smaller in size than these large-form canine *Babesia* spp. with the merozoites of *B. galileei* sp. nov. measuring on average 1.70 × 0.91 µm and the large-form canine *Babesia* spp. typically measuring 4.5–5.0 × 2.0–2.5 µm [27]. When comparing the merozoites and trophozites of *B. galileei* sp. nov. to those of other *Babesia* spp. described in cats, some differences can be noted. The most well-known feline *Babesia* sp. is *B. felis*, which was reported by Davis in 1929 from a Sudanese wild cat (*Felis ocreata* presumably *Felis sylvestris*) with a diameter of about 1.25 µm [3]. The name *B. felis* was later ascribed to a small-form *Babesia* sp. that causes severe disease in domestic cats in South Africa with an average diameter of 1.5 µm [4, 14]. *Babesia felis* described by Davis in 1929 was found in a naturally infected Sudanese wild cat kitten which did not have clinical signs of disease and maintained the infection with visible parasitemia for at least 12 months, with an episode of anemia which resolved spontaneously. The kitten’s blood was used to experimentally infect 14 cats without causing anemia or clinical disease, except for when experimentally-infected cats were splenectomized and then anemia and hemoglobinuria were detected [3]. Unfortunately, there is no known deposited specimen of this parasite and no genetic characterization of it is available. *Babesia felis* from South Africa is likely to be a different species from the one described by Davis in Sudan [3] as it appears to be somewhat larger and causes severe and potentially fatal disease in naturally as well as experimentally infected cats [14, 28, 29]. The merozoite stages of *B. galileei* sp. nov. possess abundant typical pyriform shapes, which are formed following binary fission and present as pairs of parasites attached to each other which then separate often remaining opposing each other as pear shaped couples similar in shape to those seen in large-form canine *Babesia* spp. In contrast, *B. felis* was described to rarely form pairs of pyriform or elongated merozoites, and its replication was described as the cruciform formation of four small merozoites in a tetrad shape [4, 29]. The morphology of *B. leo* is similar to that of *B. felis*, and they are also very closely related genetically [9]. *Babesia lengau* also reported from domestic cats in South Africa has merozoties measuring on average 1.91 × 1.1 µm and round to oval forms of 1.3 × 1.3 µm, and it has not been reported to form pairs of pyriform merozoites that present opposing each other [11, 12]. *Babesia hongkongensis* was described to have round to oval trophozoties with ring forms measuring 1.4 to 1.7 µm resembling small-form *Babesia* spp. with no description of pyriform merozites to date [10]. *Babesia canis* subsp. *presenti* was reported to form pairs of pyriform merozoites and also rounded trophozoites like *B. galileei* sp. nov. but these forms were larger on average than those measured for *B. galileei* sp. nov. with merozoites of 2.5 × 1.4 µm and trophozites of 2.7 × 1.7 µm compared to 1.70 × 0.91 µm and 1.53 × 1.07 µm in *B. galileei* sp. nov. [6]. *Babesia panickeri* from a domestic cat in southern India was described to have pair-shaped merozoites with average sizes of 2.7 × 1.3 µm, which is larger than those of *B. galileei* sp. nov. [13]. Lastly, *Babesia* sp. lynx genotype described from the Eurasian lynx (*Lynx lynx*) in Turkey had merozoites of 1.9 × 1 µm and trophozoites of 1.87 × 1.55 µm on average, which seem to be similar in shape but slightly bigger than those of *B. galileei* sp. nov. [30].

### Molecular phylogeny

The nearly complete piroplasmid *18S rRNA* gene (about 1400 bp) was amplified from the blood of all three cats (GenBank: PP620719-PP620721). Pairwise comparisons showed a 100% identity among the three sequences. As determined by BLAST, the closest match to the cat sequences with 99.8% identity and 97% coverage was a sequence from a *Babesia* sp. from the Eurasian lynx from Turkey termed *Babesia* sp. lynx (GenBank: MZ905342) [30] and a sequence from a *Haemaphysalis erinacei* tick also from Turkey termed *Babesia* sp. Ankara (GenBank MH504117) [31] with 99.6% identity and 98% coverage (Additional File 1). The *18S rRNA* sequences from the Israeli cats in this study were 99.2% identical (100% coverage) to *B. rossi* (GenBank KY463434), only 95.7% identical (100% coverage) to *B. canis presentii* (GenBank AY272047) and 95.5% identical (100% coverage) to *B. pisicii* (GenBank MW939360).

**Fig. 3:**
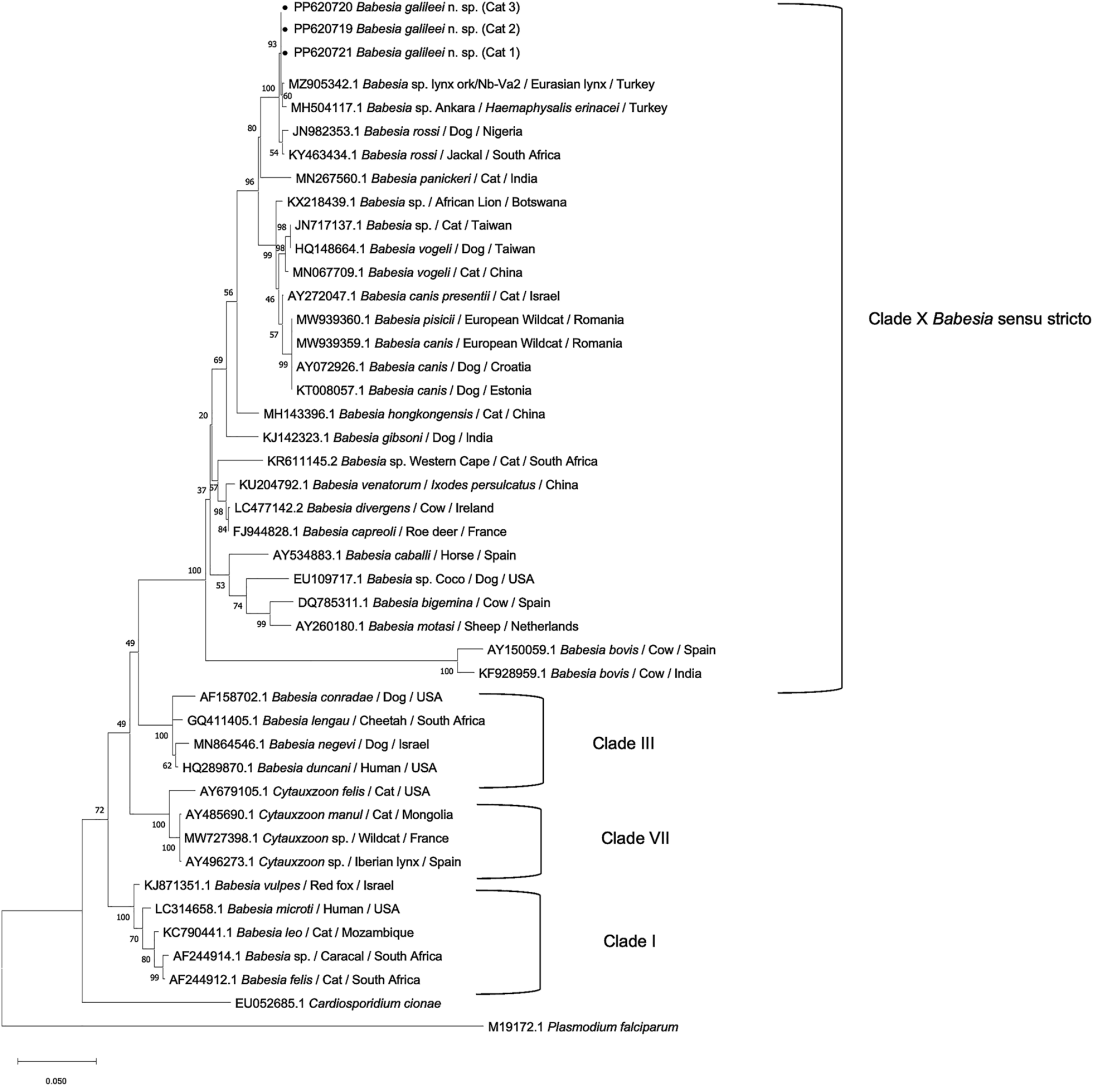
An *18S* *rRNA* phylogenetic tree based on the near full length gene. Sequences of *Babesia galileei* sp. nov. from the cats in this study (black circle) compared to other relevant piroplasmid spp. deposited in GenBank. Clade designation of piroplasmid spp. is shown according to Jalovecka et al., 2019 [32]. The GenBank accession numbers, host and country of origin are included for each sequence. The topology was inferred by using the maximum likelihood method and Tamura-Nei + G model [49]. This analysis involved 44 nucleotide sequences. All positions containing gaps and missing data were eliminated (complete deletion option). There were a total of 1295 positions in the final dataset. *Plasmodium falciparum* *18S* *rRNA* sequence was used as an outgroup. The scale bar represents the evolutionary distance in the units of the number of nucleotide substitutions per site

A phylogenetic tree inferred based on 1397-bp sequences of the *18S rRNA* gene of *B. galileei* sp. nov. and other piroplasmids present in GenBank representing a wide range of parasites of the order piroplasmida, including those that have been found to infect cats, was inferred using the maximum likelihood model (Fig. 3). Sequences of the three cats infected with *B. galileei* sp. nov. grouped within the *Babesia* s.s. clade (clade X) [32]. They clustered together with each other in a strongly supported [bootstrap (bs) 93] sister clade to *Babesia* sp. lynx (GenBank: MZ905342) and *Babesia* sp. Ankara (GenBank MH504117). These Israeli and Turkish piroplasm sequences clustered close yet significantly distinctly to *B. rossi* from African canines and *B. panickeri* from an Indian cat and then further away from *B. vogeli*, *B. canis presentii*, *Babesia pisicii*, *B. canis* and other clade X *Babesia* s.s. spp. including *B. hongkongensis* and *Babesia* sp. Western Cape from domestic cats. The Western group *Babesia* spp. (clade III), including the feline spp. *B. lengau*, formed a distinct cluster from the *Babesia* s.s. (clade X [32]), and the *Cytauxzoon* spp. infecting domestic cats and wild felines (clade VII) also clustered separately, as well as the *B. microti* group (clade I) including *B. felis* and *B. leo*, which cause disease in domestic cats in southern Africa which branched separately.

**Fig. 4:**
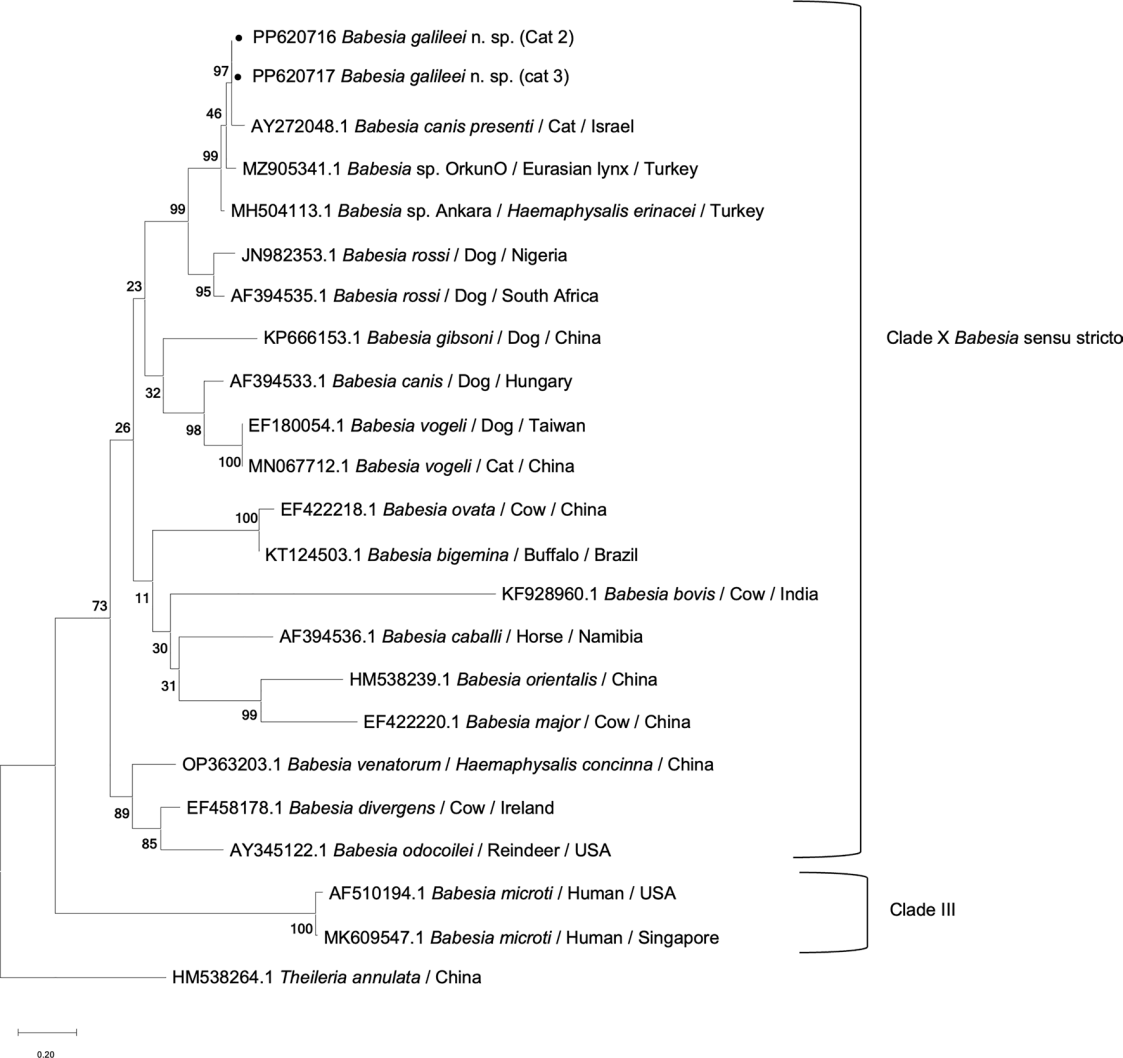
A phylogenetic tree of ITS piroplasmid sequences. Sequences of *Babesia galileei* sp. nov. from the cats in this study (black circle) are compared to other relevant piroplasmid spp. ITS sequences deposited in GenBank. Clade designation of piroplasmid spp. is shown according to Jalovecka et al., 2019 [32]. The GenBank accession numbers, host and country of origin are included for each sequence. The topology was inferred by using the maximum likelihood method and HKY + G model [23]. This analysis involved 23 nucleotide sequences. All positions with < 95% site coverage were eliminated, i.e. < 5% alignment gaps, missing data and ambiguous bases were allowed at any position (partial deletion option). There were a total of 449 positions in the final dataset. *Theileria annulata* ITS sequence was used as an outgroup. The scale bar represents the evolutionary distance in the units of the number of nucleotide substitutions per site

Analysis of a 760-bp sequence of the internal transcribed spacer (ITS) which included parts of the *18S rRNA*, ITS1 locus, *5.8S rRNA*, ITS2 locus and partial *28S rRNA* gene [33] showed that all the cats infected with *B. galileei* sp. nov. had sequences that were 98.94–100% identical to each other (GenBank: PP620716**-**PP620718**).** The closest match to the *B. galileei* sp. nov. cat sequences with 98.75% identity and 84% coverage was a sequence from *B. canis* susp. *presentii* (GenBank AY272048), followed by *Babesia* sp. Ankara (GenBank MH504112; 97% identity; 99% coverage), *Babesia* sp. lynx (GenBank MZ905341; 95% identity; 100% coverage) and *B. rossi* (GenBank AF394535; 83.3% identity; 100% coverage) (Additional File 1). Phylogenetic analysis based on a 623-bp-long sequence of the ITS (Fig. 4) showed that *B. galileei* sp. nov. and *B. canis* subsp. *presentii* clustered together and were separated with strong support (bs 85) from *Babesia* sp. Ankara, which also separated (bs 84) from *Babesia* sp. lynx. The ITS tree also showed that *B. galileei* sp. nov. was placed within the *Babesia* s.s. (clade X) and separately from the *B. microti* group (clade I).

**Fig. 5:**
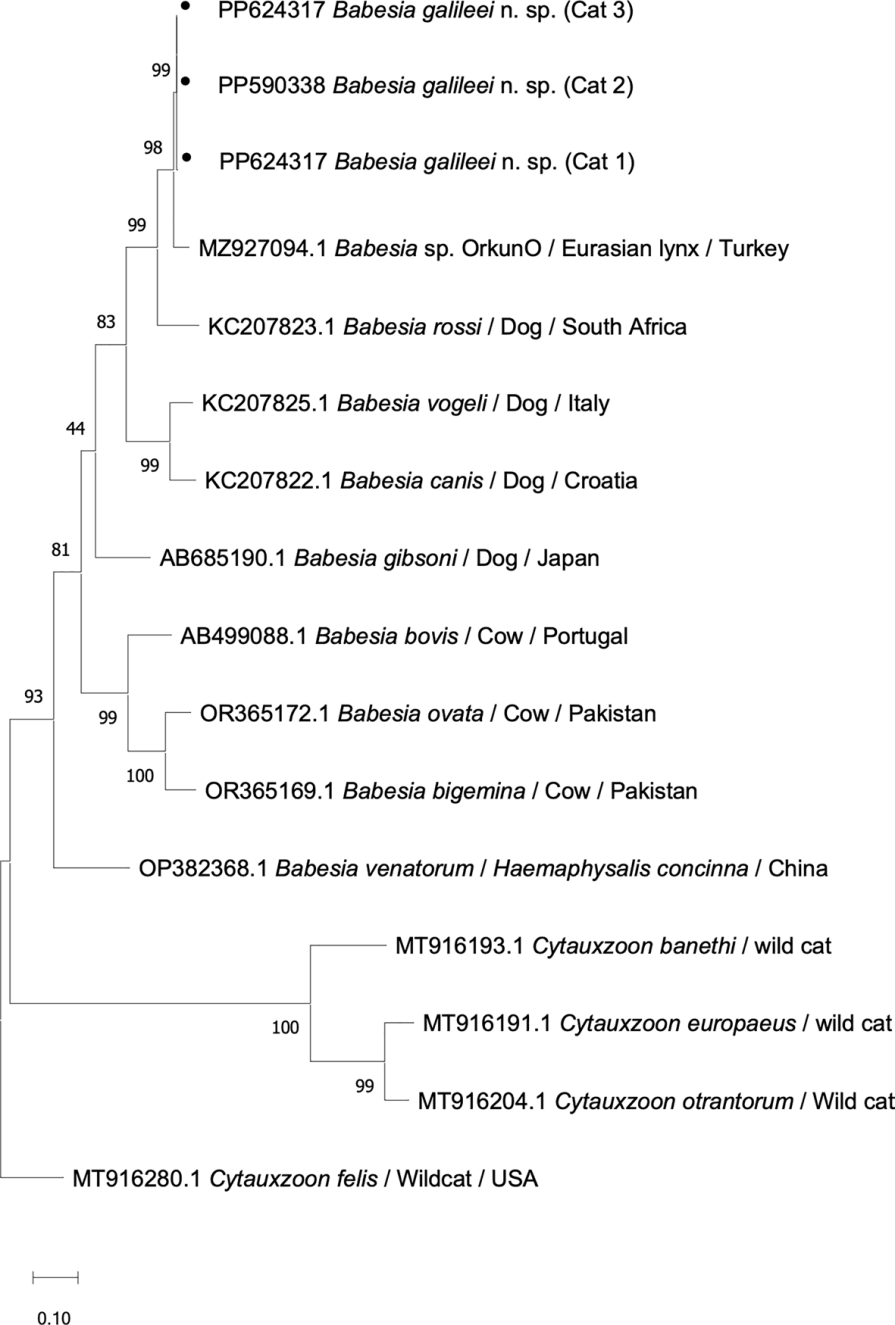
A phylogenetic tree of *CytB* gene piroplasmid sequences. Sequences of *Babesia galileei* sp. nov. from the cats in this study (black circle) are compared to other relevant piroplasmid spp. *CytB* sequences deposited in GenBank. The GenBank accession numbers, host and country of origin are included for each sequence. The topology was inferred by using the maximum likelihood method and GTR + G model [17]. This analysis involved 16 nucleotide sequences. All positions containing gaps and missing data were eliminated (complete deletion option). There were a total of 498 positions in the final dataset. *Cytauxzoon felis cytB* sequence was used as an outgroup. The scale bar represents the evolutionary distance in the units of the number of nucleotide substitutions per site

Seven hundred fifty-bp sequences of the *B. galileei* sp. nov. *CytB* gene, a mitochondrial gene, were 99.47%–100% identical to each other (GenBank PP624317, PP590338, PP590339). The closest match to the *B. galileei* sp. nov. cat sequences were *CytB* sequences from *B. pisicii* (GenBank MW93962) followed by *Babesia* sp. lynx (GenBank MZ927094; 96% identity; 100% coverage) and *B. rossi* (GenBank KC2078230) (Additional File 1). Phylogenetic analysis based on 660-bp sequences of the *CytB* gene (Fig. 5) showed that *B. galileei* sp. nov. was separated with strong support (bs 99) from *Babesia* sp. lynx and also from the canine *Babesia* s.s. spp. *B. rossi*, *B, vogeli*, *B. canis* and *B. gibsoni*, corroborating the identity of *B. galileei* sp. nov. as a novel species.

**Fig. 6:**
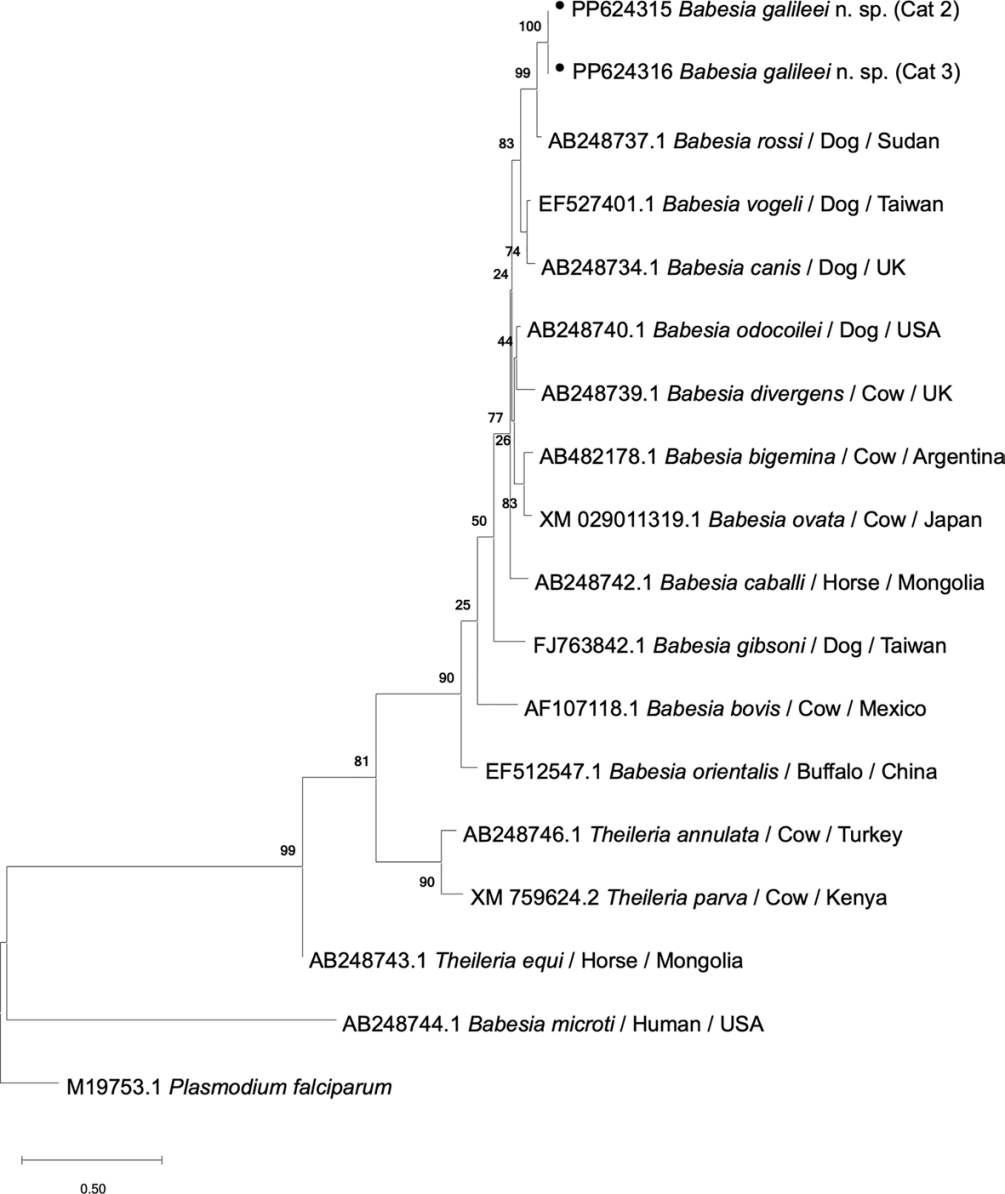
A phylogenetic tree of *HSP70* gene piroplasmid sequences. Sequences of *Babesia galileei* sp. nov. from the cats in this study (black circle) are compared to other relevant piroplasmid spp. *HSP70* sequences deposited in GenBank. The GenBank accession numbers, host and country of origin are included for each sequence. The topology was inferred by using the maximum likelihood method and Kimura two-parameter + G model [50]. This analysis involved 18 nucleotide sequences. All positions with < 95% site coverage were eliminated, i.e. < 5% alignment gaps, missing data and ambiguous bases were allowed at any position (partial deletion option). There were a total of 292 positions in the final dataset. *Plasmodium falciparim* *HSP70* sequence was used as an outgroup. The scale bar represents the evolutionary distance in the units of the number of nucleotide substitutions per site

The 330-bp *HSP70* gene sequences of *B. galileei* sp. nov. were derived only from cats # 2 and 3, as PCR for this gene from cat # 1 failed, and were 98.97% identical to each other (GenBank PP624315, PP624316). The closest match to the *B. galileei* sp. nov. HSP70 sequences was from *B. rossi* (GenBank AB248737; 95.5% identity, 100% coverage) compared to those sequenced from cat # 2 (Additional File 1). Phylogenetic analysis of a 330-bp DNA sequence of the *HSP70* gene with comparison to *HSP70* sequences of relevant *Babesia* spp. found in GenBank (Fig. 6) showed that *B. galileei* sp. nov. clustered separately with strong support (bs 100) from *B. rossi* and was also clearly separated from other *Babesia* s.s. spp. and from other species belonging to different piroplasmid clades.

Overall, the *18S rRNA* phylogeny constructed using nearly complete gene sequences and the additional analyses of the ITS locus and *CytB* and *HSP70* genes clearly demonstrated that *B. galileei* sp. nov. represents a novel distinct individual species with the *Babesia* s.s. group distinguished from other known piroplasmid spp. including all of those reported to infect felines. The DNA sequences from the Turkish lynx and *H. erinaceri* tick are the closest piroplasmid sequences to *B. galileei* sp. nov., yet they form a strongly supported separate sister clade, as shown by the *18S rRNA*, ITS and *CytB* phylogenetic analysis. *Babesia canis presentii* reported earlier from cats in Israel is also genetically distinct from *B. galileei* sp. nov., as shown by the *18S rRNA*, ITS phylogenetic analysis (Fig. 3).

### PCR of ticks

Two adult female ticks were collected from cat # 1, identified morphologically as *H. adleri* using the identification key of Feldman-Muhsam (1951) [25] and processed as a whole tick for DNA extraction and PCR. The female engorged tick was positive by *18S rRNA* PCR for *Babesia* sp. with a sequence that was 100% identical to *B. galileei* sp. nov. Eleven ticks, two adult females and nine adult males, were collected from cat # 3. All 11 ticks were identified morphologically as *H. adleri*. The female ticks from cat # 3 were dissected and were negative by PCR for *Babesia* in their salivary glands, guts and bodies. Of the male ticks from cat # 3, four were not dissected because of their small size, and DNA extracted from them was *18S rRNA* PCR-positive from one tick with a sequence of *B. galileei* sp. nov. Of the 5 male ticks dissected, three were positive by *18S rRNA* PCR, of which two were positive in the salivary glands as well as in their guts, whereas one was positive only in its guts and not in the salivary glands. Altogether, 4 of 11 (36%) *H. adelri* ticks found on cat # 3 were positive for *B. galileei* sp. nov. DNA. The presence of *B. galileei* sp. nov. DNA in the tick’s salivary gland is suggestive of the possibility that *H. adleri* could be a vector of this pathogen, as *Babesia* sporozoites develop in the salivary gland before being transferred to the vertebrae host with the tick’s saliva during a blood meal.

### Clinical findings in infected cats

The infected cats included three males from northern and central Israel.

*Cat # 1* was a 3-year-old castrated male from the rural village Kibbutz Yehiam in the Galilee region of northern Israel who was living indoors with its owners and had free access to the outdoor environment. It was admitted for veterinary medical care on 14 December 2008 in a private veterinary clinic with a history of lethargy and loss of appetite. On physical examination, it had a high rectal body temperature (40.5 ºC; normal range 38.1–39.2 ºC) and was infested with ticks. A complete blood count showed anemia [red blood cell (RBC) 3.66 × 10^6^/ul, reference interval (RI) 6–10.1 × 10^6^//ul; mean cell volume (MCV) 36.4 fL, RI 41.3–52.6 fL; mean corpuscular hemoglobin concentration (MCHC) 38.9 g/d, RI 27–32.8] and thrombocytopenia [platelets (PLT) 90 × 10^3^/ul, RI 156–626 × 10^3^/ul]. A serum biochemistry panel showed azotemia (urea 98 mg/dl RI 35–70 mg/dl; creatinine 3.5 mg/dl, RI 0.4–2 mg/dl) and increased activity of liver and muscle enzymes [alanine aminotransferase (ALT) 131 u/l, RI 15–60; aspartate transaminase (AST) 70 u/l RI 0–60]. The cat tested positive for antibodies to FIV and negative for FeLV using a commercial assay (SNAP FIV/FeLV Combo Test, IDEXX Laboratories, Westbrook, ME, USA) and was negative for *Hepatozoon*, *Anaplasma* and *Ehrlichia* spp. by PCR. A blood smear examination showed infection of RBC with piroplasmid organisms interpreted as *Babesia* sp., and blood was taken for PCR to verify and genetically characterize the infecting organism. The cat was initially treated with the antibiotic doxycycline (Dexcel Ltd., Israel) at 10 mg/kg orally once daily for 14 days and was then administered an injection of imidocarb dipropionate (Imizol; Schering-Plough, Animal Health) at 3.5 mg/kg subcutaneously once 2 days after admission when the diagnosis of babesiosis was made. It improved clinically 6 days after admission and was visited in the owner’s home 11 days after its initial admission on the 25 December 2008. The cat had recovered clinically but was infested with ticks which were collected for testing, and a second blood sample was collected for *Babesia* PCR. PCR was positive for *Babesia* sp. both before imidocarb dipropionate treatment and 9 days after the treatment. Two ticks removed from the cat were identified morphologically as *H. adleri* [25], one of which also tested PCR positive for the same *Babesia* sp. found in the cat’s blood.

*Cat # 2* was an 11-year-old castrated male from the city of Holon in central Israel that lived mostly indoors and had a history of tick infestation. It was diagnosed in a private veterinary clinic with babesiosis on 26 October 2022 after being treated for 12 days with initial lethargy, loss of appetite, dehydration, fever (40.7 ºC) and anemia. Initial blood tests on 14 October 2022 before the diagnosis of babesiosis revealed normocytic normochromic anemia (RBC 5.2 × 10^6^/ul) and thrombocytopenia (PLT 24 × 10^3^/ul). A serum biochemistry panel showed azotemia [blood urea nitrogen (BUN) 46 mg/dl RI 12–30 mg/dl; creatinine 2.2 mg/dl, RI 0.4–2 mg/dl] and increased activities of the liver enzyme ALT (147 u/l, RI 15–60), and the cat was negative for FIV and FeLV by serology (SNAP FIV/FeLV Combo Test) and for *Hepatozoon*, *Anaplasma* and *Ehrlichia* spp. by PCR. It was treated initially with doxycycline (Dexcel Ltd., Israel) at 10 mg/kg orally once daily for 14 days and then after 4 days when there was deterioration in the cat’s anemia, and it began receiving immune-suppressive therapy because of suspected immune-mediated hemolytic anemia (IMHA) with prednisolone (Rekah Pharmaceutical Products LTD, Holon, Israel) at 4 mg/kg orally once daily and after an additional day also with cyclosporine (Atopica, Novartis Animal Health, Basel, Switzerland) at 5 mg/kg q12h orally every 12 h. On day 11 after the cat’s admission, it was still anemic (RBC 4.71 × 10^6^/ul) and thrombocytopenic (PLT 79 × 10^3^/ul), and no organisms were evident on the blood smear. However, *Babesia* sp. were visualized within RBC on a blood smear prepared without performing a CBC on the following day. The cat was treated with imidocarb dipropionate at 3.5 mg/kg injected subcutaneously, and it had improved clinically and was more active on the next day, but despite this a blood smear showed *Babesia* sp. organisms which were still evident in its RBC. The cat improved gradually over the next 14 days and was negative for *Babesia* in its blood smear but still anemic (RBC 4.32 × 10^6^/ul) when administered a second imidocarb dipropionate 2 weeks after the initial injection of this drug. PCR for *Babesia* sp. was positive on the 26 October 2022 prior to the first imidocarb dipropionate injection and also a day later when *Babesia* was detected in the blood smear, but it was negative 2 weeks after the first imidocarb dipropionate treatment and before the second injection. The cat was clinically normal, and its CBC was within RI 25 days after it was diagnosed with babesiosis and received its first imidocarb dipropionate injection.

*Cat # 3* was an 11-year-old castrated male from the village of Harashim in the Galilee region of northern Israel who was living indoors with outdoor access. It was admitted to a private veterinary clinic on 8 November 2023 after disappearing from the owner’s house for 8 days and presented with extreme lethargy, loss of appetite and apparent jaundice. It was referred to the Hebrew University Veterinary Teaching Hospital (HUVTH) on the next day where on physical examination it was noted that the cat was dehydrated and thin, had yellow mucous membranes and conjunctivas, a heart murmur, a low rectal body temperature (37.2 ºC) and purulent ocular excretions and was infested with ticks. Blood tests showed anemia (RBC 4.7 × 10^6^/ul, RI 6–10.1 × 10^6^/ul; MCV 39.6 fL, RI 41.3–52.6 fL; MCHC 34.6 g/d, RI 27–32.8, leukocytosis [white blood cells (WBC) 35.9 × 10^3^/ul; RI 6.3–19.6 × 10^3^/ul] composed mainly of neutrophilia (neutrophils 28.9 × 10^3^/ul; RI 3.0–13.4 × 10^3^/ul) and monocytosis (monocytes 2.38 × 10^3^/ul; RI 0–1 × 10^3^/ul; RI) and thrombocytopenia (PLT 68 × 10^3^/ul, RI 156–626 × 10^3^/ul). A blood smear examination showed infection of RBC with piroplasmid organisms interpreted as *Babesia* sp., and blood was taken for PCR to verify and genetically characterize the infecting organism. A serum biochemistry panel showed increased creatinine (3.57 mg/dl, RI 0.4–2 mg/dl) and increased activity of ALT (82 u/l. RI 15–60) and an extremely high bilirubin level (28.5 mg/dl; RI 0–0.2 mg/dl). The cat was negative for FIV and FeLV by serology (SNAP FIV/FeLV Combo Test), negative for *Hepatozoon*, *Anaplasma* and *Ehrlichia* spp. by PCR and positive by PCR for *Candidatus* Mycoplasma haemominitum. Eleven ticks which were removed from the cat were identified morphologically as *H. adleri* and were tested thereafter for infection with *Babesia* by PCR. The cat was treated with imidocarb dipropionate at 2.5 mg/kg injected subcutaneously 1 day after its admittance to the HUVTH on 10 November 2023. No blood parasites were seen on a blood smear taken 2 days after the imidocarb dipropionate injection on the 12 November 2024; however, blood taken 2 days later on 14 November 2024 was still positive for *Babesia* sp. DNA by PCR. The cat was hospitalized at the HUVTH for 24 days with the diagnoses of babesiosis, acute kidney disease, pancreatitis and hypertophic cardiomyopathy. During hospitalization, the cat’s anemia gradually improved while its platelet and leukocyte numbers returned to the RI levels within 9 days after admission. Its bilirubin level decreased to 0.67 mg/dl with no visual icterus 17 days after hospitalization but its creatinine levels gradually increased despite fluid treatment and reached the peak of 5.25 mg/dl on 27 November 2023, after which it decreased but did not reach the RI when it was discharged. A month after discharge, the cat was treated at home daily with subcutaneous fluids because of kidney disease that became chronic and was reported to be active with good appetite despite a persistent increased creatinine level of 2.4 mg/dl.

## Discussion

This study presents *B. galileei* sp. nov. as a new taxon by fulfilling the ICZN guideline requirements for a new species [26]. The placement of *B. galileei* sp. nov. in the genus *Babesia*, and its segregation in the *Babesia* s.s. (clade x) within this genus is derived from the molecular phylogenetic analysis of the 18S *rRNA*, ITS, *CytB* and *HSP70* sequences. The phylogenetic analysis demonstrates the distinct identity of *B. galileei* sp. nov. as corroborated by the demonstration of a strongly supported clade containing the sequences of the infected domestic cats in each of the constructed phylogenetic trees. *Babesia galileei* sp. nov. is positioned as a sister clade of *Babesia* sp. lynx and *Babesia* sp. Ankara as inferred from the 18S *rRNA*, ITS and *CytB* trees. Both of the latter parasites were reported from Turkey, which is situated north of Israel in the Middle East [30, 31]. However, it should be noted that no wildlife lynx species, including the Eurasian lynx, which is described as the host of *Babesia* sp. lynx, are resident in Israel. Interestingly, both the Turkish *Babesia* genotypes and *B. galileei* sp. nov. group are closely related to *B. rossi*, a canine species, according to the 18S *rRNA*, ITS and *CytB* phylogenetic trees. *Babesia rossi* causes disease in dogs in Africa, and although it has mainly been described as a cause of severe babesiosis in domestic dogs from southern Africa, it is also present as far north in Africa as Sudan [34, 35]. The Black-backed jackal (*Canis mesomelas*) is infected sub-clinically with *B. rossi* and is considered the reservoir of this infection in South Africa [35, 36]. The presence of genetically related *Babesia* spp. along the route from Africa to Turkey, passing Israel on the way, is of evolutionary interest, as there are also animals that originated in Africa and are found following this route such as the rock hyrax (*Procavia capensis*) [37]. Therefore, the dispersion of *Babesia* spp. with migrating wildlife hosts is a likely route of parasite distribution.

The tick species *H. adleri*, which was found on cats # 1 and 3, is a palearctic *Ixodid* species that was described initially from Israel in 1951 [25] and has since then been reported also from Lebanon [38], the Palestinian Authority [39] and Iraq [40]. The vertebrate hosts of *H. adleri* include a variety of wildlife and domestic animals including mustelid, canids, suidae and felid spp. [41]. A survey of ectoparasites on cats from Jerusalem, Israel, has found that 3 of 340 cats (0.9%) were infested with *H. adleri*, and infestation was more common in the winter season [42]. Winter in Israel approaches at the end of October and continues until late March. The cases of babesiosis in the cats reported in this study, in which two cats were infested with *H. adleri*, occurred between the end of October and mid-December. This agrees with the expected period of *H. adleri*’s activity. The reported limited geographic distribution of *H. adleri*, if indeed it is the parasite’s vector, may also explain why *B. galileei* sp. nov. has not been reported so far from other distant countries except for Israel. The fact that no other species of ticks were found on the infected cats and that 36% of the ticks from cat # 3 were positive by PCR for *B. galileei* sp. nov. supports the suspicion that *H. adleri* could be the vector of this parasite. Furthermore, the detection of *B. galileei* sp. nov. DNA in the salivary glands of two ticks that were also positive in their digestive tracts strengthens the assumption that if the parasite has reached the tick’s salivary glands, it may also be transmitted by the saliva to infect a new host. However, just finding the DNA of a pathogen in a tick that has fed on blood does not indicate that this tick is capable of transmitting the pathogen. Therefore, more studies should be carried out to prove the possible capacity of *H. adleri* to serve as a vector of *B. galileei* sp. nov.

Piroplasmid spp., which infect domestic cats, are often parasites of wildlife and in particular wild felids in which they cause sub-clinical infection. Such is the case of *B. leo*, which infects lions, and *B. lengau*, whose natural host is the cheetah [9, 11, 14]. Another example is *C. felis*, which infects bobcats sub-clinically and causes a frequently fatal disease in domestic cats in North America [43]. It is likely that *B. galileei* sp. nov. is also hosted by some wildlife mammals in the Middle East, although it has not been reported in such a host yet.

The clinical presentation of the three cats with *B. galileei* sp. nov. infection includes typical findings of babesiosis including anemia, thrombocytopenia, extreme lethargy, fever in two cats and severe icterus in one cat. Co-infection with the immunosuppressive virus FIV in cat # 1 may have played a role in increasing its susceptibility to becoming infected or to progressing from infection to clinical disease. Associations between parasitic infections and FIV in populations of domestic cats have been reported previously and include an association between FIV and *Leishmania infantum* infection and a relationship between FIV and *Toxoplasma gondii* seropositivity [44, 45]. The immunosuppressive therapy that cat # 2 received with prednisolone and cyclosporine because of suspected IMHA probably contributed to the development of babesiosis in this cat, as the cat was ill before the immunosuppressive treatment began but *Babesia* organisms were detected in its blood smears only after cyclosporine treatment was begun. It is possible that the cat had been infected previously but only showed parasitemia after immunosuppression or that babesiosis was the initial cause of disease with very low parasitemia that went unnoticed in the earlier blood tests. Cat # 3 was positive for *C*. M. haemominutum; however, it is likely that this infection did not contribute to the *Babesia* infection as *C*. M. haemominutum is considered mostly non-pathogenic and has only rarely been reported as a cause of clinical disease in cats [46]. Altogether, since two cats with babesiosis included in this report were immunosuppressed by additional factors, it is plausible that immune suppression has a role in increasing the susceptibility of cats to clinical *B. galileei* sp. nov. infection.

The three cats responded well and recovered clinically following injections of imidocarb dipropionate. This agrees with *B. galileei* sp. nov. classification as *Babesia* s.s. sp. and the general responsiveness of parasites from this clade to treatment with imidocarb dipropionate, as found in dogs infected with the *Babesia* s.s. species, e.g. *B. vogeli*, *B. canis* and *B. rossi* [47], and in horses infected with *Babesia caballi* [48].

## Conclusions

This study describes a new *Babesia* sp. infecting domestic cats in the Middle East, which places phylogenetically in the *Babesia* s.s. group and is associated with severe clinical disease. Identifying new *Babesia* spp. and unraveling their clinical impact and response to drug treatment is imperative for veterinary clinicians and parasitologists. More research is warranted to confirm the tick vector of *B. galileei* sp. nov and to study other potential animal hosts for this pathogen.

### Supplementary Information

Additional file1 Table 1. Identity matches (%) of gene and loci DNA sequences from the Israeli cats compared to relevant close spp. and genotypes (% cover in brackets)

## Data Availability

All data generated or analyzed during this study are included in this published article. Analyzed nucleotide sequences used for pairwise comparisons and tree construction were submitted to the GenBank database under the accession numbers PP620719-PP620721(18S *rRNA*),PP620716-PP620718 (ITS), PP624317, PP590338, PP590339 (*CytB*) and PP624315, PP624316 (*HSP70*). The holotype was deposited in the National Natural History Collection of the Hebrew University of Jerusalem, Israel, under the accession number HUJINV 1000000006.

## Abbreviations

HSP: Heat shock protein
ICZN: International code of zoological nomenclature
ITS: Internal transcribed spacer
PCR: Polymerase chain reaction
s.s.: Sensu stricto
