# Supplementary material for: Description of Babesia galileei sp. nov. A piroplasmid species causing severe disease in domestic cats
Source: Parasit Vectors. 2024 Jul 9;17:297. doi: 10.1186/s13071-024-06371-w (PMC11234728; doi:10.1186/s13071-024-06371-w)
Supplement: Supplementary file 1 — Additional file1 Table 1. Identity matches (%) of gene and loci DNA sequences from the Israeli cats compared to relevant close spp. and genotypes (% cover in brackets) [file 13071_2024_6371_MOESM1_ESM.pptx]

## Slide 1
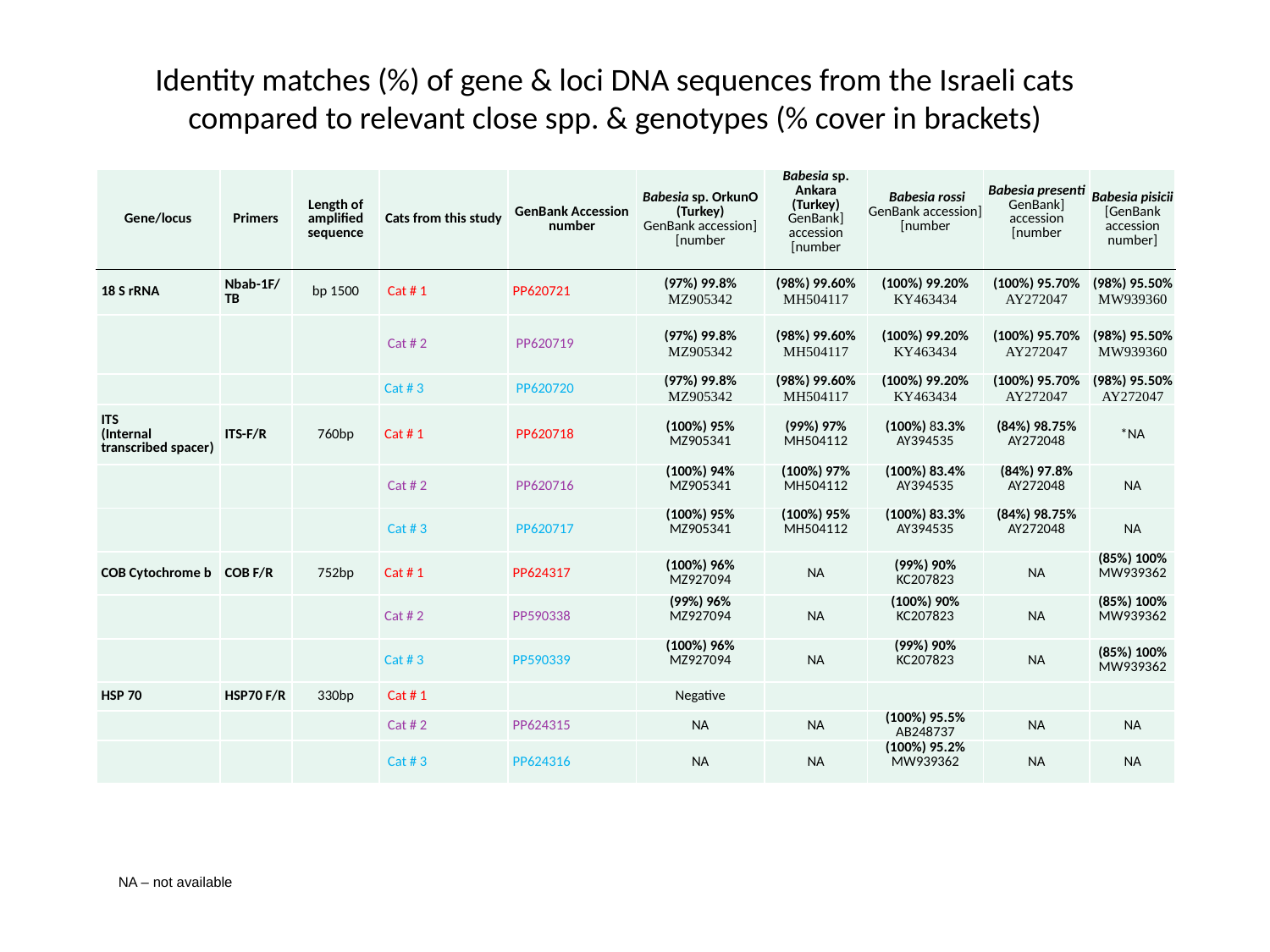

Identity matches (%) of gene & loci DNA sequences from the Israeli cats compared to relevant close spp. & genotypes (% cover in brackets)
| Gene/locus | Primers | Length of amplified sequence | Cats from this study | GenBank Accession number | Babesia sp. OrkunO (Turkey) [GenBank accession number] | Babesia sp. Ankara (Turkey) [GenBank accession number] | Babesia rossi [GenBank accession number] | Babesia presenti [GenBank accession number] | Babesia pisicii [GenBank accession number] |
| --- | --- | --- | --- | --- | --- | --- | --- | --- | --- |
| 18 S rRNA | Nbab-1F/TB | 1500 bp | Cat # 1 | PP620721 | 99.8% (97%) MZ905342 | 99.60% (98%) MH504117 | 99.20% (100%) KY463434 | 95.70% (100%) AY272047 | 95.50% (98%) MW939360 |
| | | | Cat # 2 | PP620719 | 99.8% (97%) MZ905342 | 99.60% (98%) MH504117 | 99.20% (100%) KY463434 | 95.70% (100%) AY272047 | 95.50% (98%) MW939360 |
| | | | Cat # 3 | PP620720 | 99.8% (97%) MZ905342 | 99.60% (98%) MH504117 | 99.20% (100%) KY463434 | 95.70% (100%) AY272047 | 95.50% (98%) AY272047 |
| ITS (Internal transcribed spacer) | ITS-F/R | 760bp | Cat # 1 | PP620718 | 95% (100%) MZ905341 | 97% (99%) MH504112 | 83.3% (100%) AY394535 | 98.75% (84%) AY272048 | NA\* |
| | | | Cat # 2 | PP620716 | 94% (100%) MZ905341 | 97% (100%) MH504112 | 83.4% (100%) AY394535 | 97.8% (84%) AY272048 | NA |
| | | | Cat # 3 | PP620717 | 95% (100%) MZ905341 | 95% (100%) MH504112 | 83.3% (100%) AY394535 | 98.75% (84%) AY272048 | NA |
| COB Cytochrome b | COB F/R | 752bp | Cat # 1 | PP624317 | 96% (100%) MZ927094 | NA | 90% (99%) KC207823 | NA | 100% (85%) MW939362 |
| | | | Cat # 2 | PP590338 | 96% (99%) MZ927094 | NA | 90% (100%) KC207823 | NA | 100% (85%) MW939362 |
| | | | Cat # 3 | PP590339 | 96% (100%) MZ927094 | NA | 90% (99%) KC207823 | NA | 100% (85%) MW939362 |
| HSP 70 | HSP70 F/R | 330bp | Cat # 1 | | Negative | | | | |
| | | | Cat # 2 | PP624315 | NA | NA | 95.5% (100%) AB248737 | NA | NA |
| | | | Cat # 3 | PP624316 | NA | NA | 95.2% (100%) MW939362 | NA | NA |
NA – not available
